# Lactic Acid and Glutamine Have Positive Synergistic Effects on Growth Performance, Intestinal Function, and Microflora of Weaning Piglets

**DOI:** 10.3390/ani14233532

**Published:** 2024-12-06

**Authors:** Junjie Jiang, Daiwen Chen, Bing Yu, Jun He, Jie Yu, Xiangbing Mao, Zhiqing Huang, Yuheng Luo, Junqiu Luo, Ping Zheng

**Affiliations:** Key Laboratory for Animal Disease-Resistance Nutrition of China Ministry of Education, Institute of Animal Nutrition, Sichuan Agricultural University, Chengdu 611130, China; jiangjj04@tongwei.com (J.J.); chendwz@sicau.edu.cn (D.C.); ybingtian@163.com (B.Y.); hejun8067@163.com (J.H.); yujie@sicau.edu.cn (J.Y.); acatmxb2003@163.com (X.M.); zqhuang@sicau.edu.cn (Z.H.); luoluo212@126.com (Y.L.); junqluo2018@tom.com (J.L.)

**Keywords:** weaning piglet, lactic acid, glutamine, growth performance, intestinal function, microflora

## Abstract

The objective of this study was to evaluate the effects of dietary addition of lactic acid and glutamine, and their interactions, on growth performance, nutrient digestibility, digestive enzyme activity, intestinal barrier functions, microflora, and expressions of intestinal development-related genes of weaning piglets. Ninety-six 24-day-old weaning piglets (Duroc × Landrace × Yorkshire, weaned at 21 ± 1 d and fed the basal diet for a 3 d adaptation period) with initial body weight of 7.24 ± 0.09 kg were randomly assigned to one of four dietary treatments with six replicates per treatment and four pigs per replicate in a 2 × 2 factorial treatment arrangements: (1) CON (a 2-period basal diet; control), (2) LS (supplemented with 2% lactic acid), (3) GS (supplemented with 1% glutamine), and (4) LGS (supplemented with 2% lactic acid and 1% glutamine). The study lasted for 28 d. On days 25–28, fresh fecal samples were collected to evaluate apparent total tract digestibility (ATTD) of nutrients. After 28 d, one weaning pig per pen was euthanized, and physiological samples obtained. Results showed that the supplementation of lactic acid improved the ADFI of the pigs (*p* < 0.05), while the pigs fed the glutamine diet had a greater ADFI and higher G/F (*p* < 0.05), and there were significant interactive effects between lactic acid and glutamine on the ADFI and G/F of the pigs (*p* < 0.05). The ATTD of CP and ash for pigs fed with lactic acid was significantly enhanced, and pigs fed the glutamine diet had greater ATTD of CP and ash (*p* < 0.05), while there were significant interactive effects between lactic acid and glutamine on the ATTD of CP and ash of the pigs (*p* < 0.05). Pigs fed with lactic acid exhibited greater activity of α-amylase and lipase (*p* < 0.05); moreover, the activity of lipase in the pigs showed a significant interactive effect between lactic acid and glutamine (*p* < 0.05). There was a greater villus height and villus height to crypt depth ratio in pigs fed with lactic acid (*p* < 0.05), and the villus height to crypt depth ratio of pigs fed with glutamine was greater (*p* < 0.05). There were greater *GLUT2*, *IGF-1*, *TGF-β2*, *OCLN*, and *ZO-1* mRNA levels in pigs fed with lactic acid (*p* < 0.05), and the supplementation of glutamine increased *SGLT1*, *GLUT2*, *PepT1*, *IGF-1*, *IGF-1R*, *TGFβ-2*, *GLP-2*, and *OCLN* mRNA levels (*p* < 0.05), Additionally, expressions of *SGLT1*, *GLUT2*, *PepT1*, *IGF-1*, *IGF-1R*, *TGFβ-2*, *GLP-2*, *CLDN-2*, *OCLN*, and *ZO-1* mRNA levels of pigs showed a positive interactive effect between lactic acid and glutamine (*p* < 0.05). Supplementation of lactic acid significantly increased the populations of *Bifidobacterium* in cecal digesta, *Lactobacillus* in colonic digesta, and the content of butyric acid in colonic digesta (*p* < 0.05). In addition, there were significant interactive effects between lactic acid and glutamine on populations of *Bifidobacterium* in cecal digesta, *Lactobacillus* in colonic digesta, and the content of acetic acid, butyric acid, and total VFAs in cecal digesta of the pigs (*p* < 0.05). Collectively, the current results indicate that dietary supplementation with lactic acid and glutamine had a positive synergistic effect on weaning pigs, which could improve growth performance through promoting the development of the small intestine, increasing digestive and barrier function, and regulating the balance of microflora in pigs, and which might be a potential feeding additive ensemble to enhance the health and growth of weaning piglets in the post-antibiotic era.

## Simple Summary

In China, following the ban on feed antibiotics in 2020, the intestinal health problems of weaning piglets have become increasingly serious; however, diets supplemented with a single dietary additive for weaning piglets cannot always solve adverse problems caused by weaning. Lactic acid promotes the activity of digestive enzymes and improves the subsequent digestion of young piglets by regulating gastrointestinal pH, and it can also play an important role as an energy substance in the tricarboxylic acid cycle. Glutamine, as the most abundant free amino acid in animal organisms, can restore the defective intestinal mucosal structure and immune function caused by weaning. Dietary supplementation with lactic acid and glutamine could be a strategy for alleviating the weaning stress of piglets; however, it is unknown whether the combined addition of these has an interactive effect. In this study, we aimed to investigate the effects of dietary supplementation with lactic acid and glutamine, and their interactions, on growth performance, intestinal function, and microflora of weaning piglets.

## 1. Introduction

Weaning stressors, such as society (physical and psychological separation), environment (transportation), and diet (form and source change of diets), widely exist during the post-weaning period [1,2,3] and are associated with destroyed intestinal function, including destruction of intestinal morphology and barrier, insufficient secretion of enzyme activity, and disruption in the stability of intestinal microflora [4,5,6], resulting in severe diarrhea, inadequate nutrient deposition, and, subsequently, unsatisfactory performance of the pigs [7,8]. In China, following the ban on feed antibiotics in 2020, the intestinal health problems of weaning piglets have become increasingly serious, causing significant economic losses [9,10], which has led to pressure to search for alternative materials or methods to replace antibiotics in the animal industry. However, diets supplemented with a single dietary additive on weaning piglets cannot always solve adverse problems caused by weaning [11]. Therefore, in recent years, scientists have studied whether the combination of certain additives with different mechanisms might offer a viable and practical method to prevent weaning stress syndrome in the post-antibiotic era.

Lactic acid, as a kind of common organic acid, was proven to be a growth-promoting agent in weaning piglets [12], and has been repeatedly shown to have beneficial effects on intestinal health and growth performance in weaning piglets [13,14]. Recently, scientists have shown that lactic acid could not only prevent and even cure intestinal inflammation in mice and humans as a bio-active molecule, but also play an essential role in the tricarboxylic acid cycle as an energy supplier [15,16]. Glutamine, as the most abundant free amino acid in animal organisms, has been used in feed and food additives for decades [17]. Relevant studies have reported that glutamine improves health status and growth performance of piglets by protecting the integrity and function of intestine [18]. In the feed industry, the combination of multiple feed additives is a common practice [19]; it is worth studying whether the combination of additives with similar functions has positive synergistic effects. Lactic acid and glutamine are both common additives in the feed industry, which improve the digestive and absorptive function of the gastrointestinal tract of weaning piglets through their respective special mechanisms. However, little is known about whether the combined supplementation of lactic acid and glutamine in the diet has a positive synergistic effect in preventing the intestinal stress induced by weaning and subsequent growth retardation in weaned piglets.

The present experiment was designed to evaluate the effects of dietary addition of lactic acid and glutamine, and their interactions, on growth performance, nutrient digestibility, digestive enzyme activity, intestinal barrier functions, microflora, and expressions of intestinal development-related genes of weaning piglets, and has provided new insights into appropriate applications of lactic acid and glutamine for the pig industry in the post-antibiotic era.

## 2. Materials and Methods

The primary experiment was conducted at the Animal Experiment Center of Sichuan Agricultural University, Ya’an, China. All animal procedures associated with this study were approved by the Animal Care and Use Committee, Sichuan Agricultural University (Ethical Approval Code: SICAUAC201906-4; Ya’an, China).

The lactic acid (≥80% purity, manufacturer’s specifications) and glutamine (≥99.5% purity, manufacturer’s specifications) were provided by Henan Jindan Lactic Acid Technology Co., Ltd., Zhoukou, China and Xinjiang Fufeng Biology Technology Co., Ltd., Urumqi, China.

### 2.1. Experimental Design and Animal Management

A total of ninety-six 24-day-old pigs (Duroc × Landrace × Yorkshire, weaned at 21 ± 1 d and fed the control diet for a 3 d adaptation period) with body weight (BW) of 7.24 ± 0.09 kg were used in a 28 d experiment. At the beginning of the experiment, pigs were randomly assigned to 4 treatments with 6 replicate pens (2 males and 2 females per pen) on the basis of their initial BW and sex in a 2 × 2 factorial treatment arrangements. The 4 dietary treatment groups comprised (1) CON (a 2-period basal diet; control), (2) LS (supplemented with 2% lactic acid), (3) GS (supplemented with 1% glutamine), and (4) LGS (supplemented with 2% lactic acid and 1% glutamine). The basal diet (Table 1) was formulated based on NRC (2012) recommendations [20].

All the experimental pigs with water ad libitum were fed the diets three times per day at 08:00, 14:00, and 20:00 h, and housed in an identical room with controlled temperature (27 ± 1 °C) and relative humidity (55% to 65%).

At the beginning and the end of the experiment, after 12 h of fasting, all weaning pigs were weighed, and the average daily gain (ADG), average daily feed intake (ADFI), and gain to feed ratio (G/F) were calculated using feed intake (FI) per pen daily throughout the entire experiment.

During the experiment, from d 1 to d 28, the occurrence of diarrhea among the four piglets per pen was observed and recorded by the same person every morning and evening continuously, and an accumulative diarrhea score per treatment and day was determined to calculate the diarrhea rate [21]: diarrhea rate (%) = D/(T × 28 d) × 100, in which D = total number of diarrheal pigs per pen and T = number of pigs per pen.

### 2.2. Sampling

The dry basal diet samples and fresh fecal samples collected using a partial collection method per pen were collected for chemical analysis after defecation from d 25 to d 28 during the experiment. Next, 10 mL of 10% dilute sulfuric acid solution was applied to each 100 g of fecal samples in plastic bags for the preservation and fixation of fecal nitrogen, and which was stored for detection at −20 °C.

On day 29 at 09:00 h, one weaning piglet per pen with average BW was euthanized with sodium pentobarbital [22], accompanied by abdominal incision; the intestine sections were collected gently, and then the entire small intestine was removed, cleaned, and cut into 3 sections (duodenum, jejunum, and ileum) [23]. Using the following 3 steps, approximately 1 cm, 2 cm, and 3 cm segments of proximal duodenum, jejunum, and ileum, respectively, were collected as follows: (1) immediately separate, wash with 0.9% physiological saline, and store in 10% formaldehyde–phosphate buffer for morphology analysis; (2) gently scrape the jejunal mucosa on an icy surface, and store at −80 °C for RNA extraction and enzyme activities; and (3) collect and store the digesta from the middle cecum (10 cm) and middle colon (10 cm) at −80 °C for measuring microbial quantity and microbial metabolites.

### 2.3. Apparent Digestibility of Nutrients and Enzyme Activities

The fresh feces from d 25 to d 28 during the experiment from each pen were mixed thoroughly with cleaned plastic bags, dried at 65 °C for 72 h, and then ground to pass through a 40-mesh screen. Acid-insoluble ash as an endogenous indicator was applied to measure the apparent total tract digestibility (ATTD) of nutrients, which was analyzed using the Chinese National Standard Method (GB/T 23742) [24]. All samples were analyzed for dry matter (method 930.15; AOAC, 1995), ash (method 923.03; AOAC, 1995), crude fat (method 920.39; AOAC, 1995), and crude protein (method 990.03; AOAC, 1995) [25], and specific adiabatic oxygen bomb calorimetry (Parr Instrument Co., Moline, IL, USA) was employed for the determination of gross energy. The following formula was used to calculate ATTD: ATTD (%) = {1 − [(A_1_ × F_2_)/(A_2_ × F_1_)]} × 100, in which A_1_ = dietary AIA content, A_2_ = fecal AIA content, F_1_ = dietary nutrient content, and F_2_ = fecal nutrient content.

The jejunal sample was pretreated before measuring the activity of enzymes; the pretreatment procedure was according to the following 2 steps: (1) the sample was weighed and homogenized with pre-cooled physiological saline according to a mass volume ratio of 1:9 (g/mL), and (2) the acquired mixture was centrifuged at 4000× *g* for 10 min at 4 °C, and then the supernatant solution was obtained. Supernatant protein concentration and the activities of trypsin, lipase, amylase, lactase, maltase, and sucrase were determined with commercial kits provided by Nanjing Jiancheng Bioengineering Institute, Nanjing, China.

### 2.4. Intestinal Morphology

The jejunum morphology was measured in line with the previous study [26]. Briefly, the jejunum sample was fixed in neutral buffered formaldehyde, dehydrated, and embedded in paraffin, and then made into 5 μm slices, followed by staining with hematoxylin and eosin. An Olympus CK 40 microscope, provided by Olympus Optical Company, Shenzhen, China, was applied to measure villus height and crypt depth and to calculate villus height to crypt depth ratio.

### 2.5. Real-Time Quantitative PCR

0.1 g of jejunum mucosal samples was homogenized in 1 mL RNAiso Plus reagent provided by TaKaRa, Dalian, China, after which the total RNA of the samples was extracted and stored at −80 °C according to the instructions. The quality and concentration of total RNA were determined using a nucleic acid protein instrument, which measured the corresponding optical density (OD) ratio of the OD_260_ to OD_280_ ratio ranging from 1.8 to 2.0. Moreover, a PrimeScript^TM^ reverse transcription reagent kit provided by TaKaRa, Dalian, China was used to synthetize cDNA.

Specific primers (Table 2) for the sodium–glucose cotransporter 1 (*SGLT1*), glucose transporter 2 (*GLUT2*), peptide transporter 1 (*PePT1*), insulin-like growth factor 1 (*IGF-1*), insulin-like growth factor 1 receptor (*IGF-1R*), transforming growth factor-β2 (*TGF-β2*), glucagon-like peptide 2 (*GLP-2*), claudin 1 (*CLDN-1*), claudin 2 (*CLDN-2*), occludin (*OCLN*), zonula occludens 1 (*ZO-1*), and zonula occludens 2 (*ZO-2*) were designed and purchased from Invitrogen (Shanghai, China). The real-time PCR reactions with SYBR Green PCR reagents provided by TaKaRa, Dalian, China were performed on a Real-Time PCR Detection System (Bio-Rad Laboratories, Inc., Hercules, CA, USA), after which a melting curve analysis was generated to check and verify the specificity and purity of all PCR products, and the reference gene transcript (β-actin) was chosen as the reference gene to normalize cDNA loading. After verification that the primers amplified with an efficiency of 100%, the results were determined using the 2^−ΔΔCt^ method [27].

### 2.6. Microbial Metabolite Analysis

0.7 g of cecal and colonic digesta samples was used to analyze the concentration of volatile fatty acids (VFA) according to the following 3 steps: (1) the digesta samples were weighed and homogenized with pre-cooled physiological saline according to a mass volume ratio of 1:1 (g/mL), and the supernatants of the digesta samples were centrifuged at 500× *g* for 10 min; (2) 2 mL of the supernatant was centrifuged at 12,000× *g* for 10 min, after which 1 mL of the supernatant was transferred, to which 0.2 mL of 25% metaphosphoric acid was added, and then centrifuged at 12,000× *g* for 10 min; and (3) 500 μL of the supernatant was transferred, to which 500 μL of methanol was added, and then the mixture was centrifuged at 12,000× *g* for 10 min, with the resulting supernatant being stored at −20 °C prior to determining the VFAs including acetic acid, propionic acid, and butyric acid with a gas chromatographic system provided by Varian, Palo Alto, CA, USA.

### 2.7. Statistical Analysis

All data (*n* = 6) were analyzed by means of a two-way ANOVA using the Generalized Linear Models procedure of SAS 9.0 software (SAS, Raleigh, NC, USA) according to the 2 × 2 factorial treatment arrangements. For the performance and digestibility trial, the pen was taken as the experimental unit, while for other parameters, individual pigs were taken as the experimental unit. The statistical model included lactic acid, glutamine, and their interactive effects. The results are presented as means and SEM. Statistical differences among treatments were determined by Tukey’s Honestly Significant Difference (HSD) test. For significance determination, the data results were significant with *p* < 0.05, and 0.05 < *p* < 0.1 was considered as a tendency. No covariates or blocking factors were applied.

## 3. Results

### 3.1. Growth Performance

No deaths occurred among pigs in the 4 groups throughout the trial. The supplementation of lactic acid significantly improved the ADFI of the pigs (*p* < 0.05). The pigs fed the glutamine diet had a greater ADFI and higher G/F (*p* < 0.05). Moreover, there were significant interactive effects between lactic acid and glutamine on the ADFI and G/F of the pigs (*p* < 0.05) (Table 3).

### 3.2. Apparent Total Tract Digestibility

As shown in Table 4, the ATTD of CP and ash for the pigs fed with lactic acid was significantly enhanced (*p* < 0.05). The pigs fed the glutamine diet had greater ATTD of CP and ash (*p* < 0.05). Furthermore, there were significant interactive effects between lactic acid and glutamine on the ATTD of CP and ash in the pigs (*p* < 0.05).

### 3.3. Digestive Enzyme Activities

The pigs fed with lactic acid exhibited greater activities of α-amylase and lipase in the jejunum (*p* < 0.05). The activities of α-amylase and lipase in the jejunum of the pigs fed with glutamine tended to be greater (*p* < 0.10). In addition, the activities of lipase in the jejunum of the pigs showed significant interactive effects between lactic acid and glutamine (*p* < 0.05) (Table 5).

### 3.4. Intestinal Morphology

**Figure 1:**
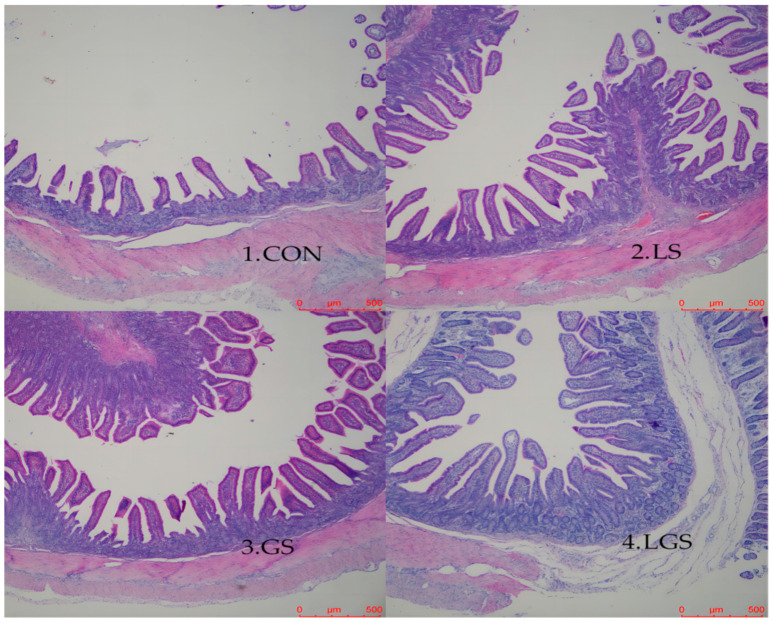
Comparison of jejunal microscopic photographs with histological staining of weaning piglets. 1. CON, the basal diet; 2. LS, supplemented with 2% lactic acid; 3. GS, supplemented with 1% glutamine; 4. LGS, supplemented with 2% lactic acid and 1% glutamine.

The jejunal morphology is shown in Table 6 and Figure 1. There was a greater villus height and villus height to crypt depth ratio in the jejunum of the pigs fed with lactic acid (*p* < 0.05). The villus height to crypt depth ratio in the jejunum of the pigs fed with glutamine was greater (*p* < 0.05), whereas there was no significant interactive effect between lactic acid and glutamine on the jejunal morphology of the pigs.

### 3.5. mRNA Levels of Nutrient Transporter-, Barrier-, and Development-Related Genes

**Figure 2:**
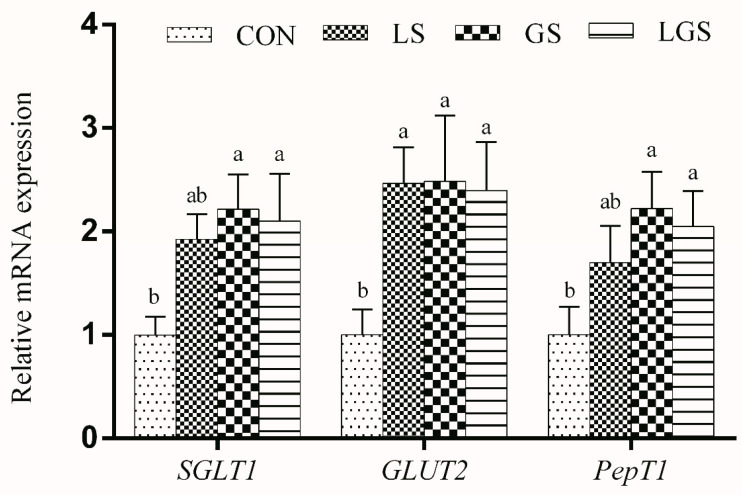
Effects of lactic acid, glutamine, and their interactions on the mRNA levels of jejunal nutrient transporter-related genes in weaning piglets. Each column represents the mean expression level of six independent replications. Letters above the bars (a, b) indicate statistical significance (*p* < 0.05) of gene expression among the four treatments. CON, the basal diet; LS, supplemented with 2% lactic acid; GS, supplemented with 1% glutamine; LGS, supplemented with 2% lactic acid and 1% glutamine; *SGLT1*, sodium–glucose cotransporter 1; *GLUT2*, glucose transporter 2; *PePT1*, peptide transporter 1.

The mRNA expression levels of intestinal nutrient transporters (*SGLT1*, *GLUT2*, and *PepT1*) in the jejunum of the pigs are shown in Figure 2. There were greater *GLUT2* mRNA levels in the jejunum of the pigs fed with lactic acid (*p* < 0.05). The supplementation of glutamine increased *SGLT1*, *GLUT2*, and *PepT1* mRNA levels in the jejunum of the pigs (*p* < 0.05). Additionally, expressions of *SGLT1*, *GLUT2*, and *PepT1* mRNA levels in the pigs showed a positive interactive effect between lactic acid and glutamine (*p* < 0.05).

**Figure 3:**
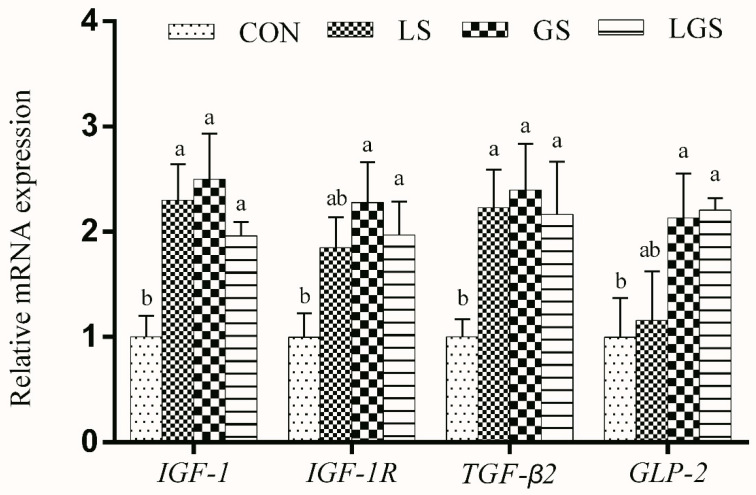
Effects of lactic acid, glutamine, and their interactions on the mRNA levels of jejunal development-related genes in weaning piglets. Each column represents the mean expression level of six independent replications. Letters above the bars (a, b) indicate statistical significance (*p* < 0.05) of gene expression among the four treatments. CON, a basal diet; LS, supplemented with 2% lactic acid; GS, supplemented with 1% glutamine; LGS, supplemented with 2% lactic acid and 1% glutamine; *IGF-1*, Insulin- like growth factor 1; *IGF-1R*, Insulin- like growth factor 1 receptor; *TGF-β2*, Transforming growth factor β2; *GLP-2*, Glucagon-like peptide 2.

*IGF-1* and *TGF-β2* mRNA levels in the jejunum of the pigs fed with lactic acid were significantly higher (*p* < 0.05). There were greater *IGF-1*, *IGF-1R*, *TGFβ-2*, and *GLP-2* mRNA levels in the pigs fed with glutamine (*p* < 0.05). There were significant interactive effects between lactic acid and glutamine on expressions of *IGF-1*, *IGF-1R*, *TGFβ-2*, and *GLP-2* mRNA levels in the pigs (*p* < 0.05) (Figure 3).

**Figure 4:**
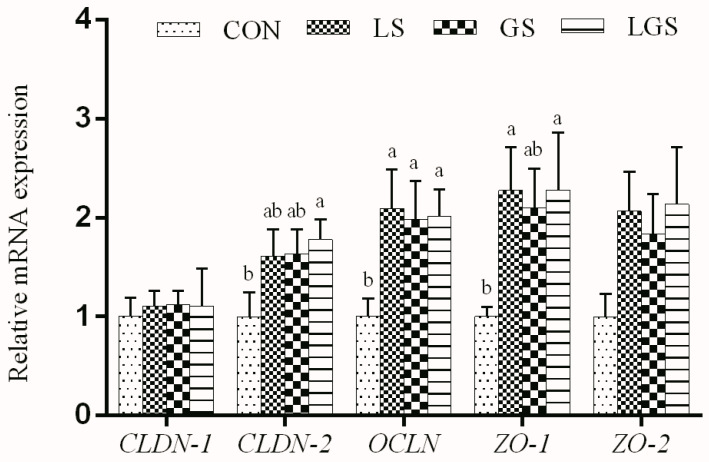
Effects of lactic acid, glutamine and their interactions on the mRNA levels of jejunal barrier-related genes in weaning piglets. Each column represents the mean expression level of six independent replications. Letters above the bars (a, b) indicate statistical significance (*p* < 0.05) of gene expression among the four treatments. CON, a basal diet; LS, supplemented with 2% lactic acid; GS, supplemented with 1% glutamine; LGS, supplemented with 2% lactic acid and 1% glutamine; *CLDN-1*, claudin 1; *CLDN-2*, claudin 2; *OCLN*, occludin; *ZO-1*, zonula occludens 1; *ZO-2*, zonula occludens 2.

As shown in Figure 4, *OCLN* and *ZO-1* mRNA levels in the jejunum of pigs fed with lactic acid were significantly higher (*p* < 0.05). The supplementation of glutamine had a significant positive effect on *OCLN* mRNA expression (*p* < 0.05). Moreover, expressions of *CLDN-2*, *OCLN*, and *ZO-1* mRNA levels of pigs showed positive interactive effects between lactic acid and glutamine (*p* < 0.05).

### 3.6. Gut Microbiome

Supplementation of lactic acid significantly increased *Bifidobacterium* populations in cecal digesta and *Lactobacillus* populations in colonic digesta (*p* < 0.05). In addition, there were significant interactive effects between lactic acid and glutamine on the populations of *Bifidobacterium* in cecal digesta and *Lactobacillus* populations in colonic digesta of the pigs (*p* < 0.05) (Table 7).

### 3.7. Microbial Metabolites

Table 8 presents the differences in intestinal microbial metabolites between the four groups. Supplementation of lactic acid significantly increased the content of butyric acid in colonic digesta (*p* < 0.05). Moreover, there were significant interactive effects between lactic acid and glutamine on acetic acid, butyric acid, and total VFAs in cecal digesta of the pigs (*p* < 0.05).

## 4. Discussion

Recent studies have shown that lactic acid or glutamine has positive effects on the growth performance and intestinal health of pigs via their specific actions [13,28,29], whereas the effects of the combined addition of lactic acid and glutamine on the performance and intestinal health of weaning piglets are still unknown. Previous studies have shown that a diet supplemented with 480 mg/kg of calcium lactate improved the growth performance of weaning pigs [30], and piglets fed a diet supplemented with 0.2% lactic acid showed improved ADG and ADFI by 15% and 12%, respectively [28]. Moreover, previous studies have reported that dietary 1% glutamine supplementation had a higher ADG and G/F in weaning pigs [31,32]. Similar findings were observed by other researchers [33,34], and they reported that glutamine could prevent intestinal dysfunction after weaning by regulating antioxidant function, thereby improving the growth performance of weaning piglets. In the present study, the supplementation of lactic acid improved the ADFI of the pigs, and the pigs fed the glutamine diet had a greater ADFI and higher G/F. Meanwhile, performance data from the current study revealed that there were positive interactive effects between lactic acid and glutamine on the ADFI and G/F of pigs. There was no published research on the effects of the lactic acid and glutamine combination on growth performance in weaning piglets. The beneficial effects of the lactic acid and glutamine combined supplementation on the ADFI and G/F of the weaning piglets might be associated with synergistic effects between lactic acid and glutamine. Lactic acid could promote the absorption and digestion of nutrients and improve the microecology balance, thus improving performance of piglets [13]. Meanwhile, glutamine improved intestinal barrier, absorptive, and digestive functions by supplying energy and precursors for intestinal epithelial cells [31,32,33,34,35]. Thus, we suspected that lactic acid and glutamine had a synergistic effect in the gut (especially on intestine epithelial cells) due to the better gut characteristics, including preferential digestibility, superior microenvironment, and better morphology, which led to better growth performance in the weaning piglets [13,28,29]. A previous study showed that compound organic acid (containing lactic acid) increased the ATTD of crude fiber in weaning piglets [36], while supplementation with 0.3% lactic acid stimulated the AID of amino acids in growing–finishing pigs [37]. A previous study found that the ATTD of dry matter and ash were significantly enhanced by organic acid (containing lactic acid) in weaning piglets [38], which is consistent with our results that the ATTD of CP and ash for pigs fed with lactic acid was significantly enhanced. In addition, in this study, we found that pigs fed the glutamine diet had greater ATTD of CP and ash. These results are in line with the previous study [31], which reported that glutamine supplementation could increase the ATTD of dry matter and CP in weaning piglets. Moreover, in our study, there were significant interactive effects between lactic acid and glutamine on the ATTD of CP and ash in pigs. We suspected that the possible reason for this was due to the synergistic effects between lactic acid and glutamine [39,40,41,42]. Organic acid supplementation could decrease gut pH and improve structure and function in the gut, resulting in enhancing the utilization rate of energy and amino acid [39,40]. Moreover, glutamine supplementation also improved the structure and function of the gut, thus increasing the digestive and absorptive functions of the gut [41,42]. Collectively, synergistic effects of lactic acid and glutamine might result in better ATTD of nutrients; consistent with this, we found that pigs showed positive interactive effects between lactic acid and glutamine on the activities of digestive enzymes and expressions of nutrient transporters in the jejunum. The enzyme activities in the digestive tract were considered as important factors that would influence intestinal health and nutrient digestibility [43]. Proper acidity in the gut of piglets is one of the most important elements for the digestive system, as pH can affect the intestinal digestive enzyme activities and microflora of digesta, and organic acid supplementation could decrease gut pH [44]. Compound organic acids could increase digestive enzyme activities in the jejunum [45], which is consistent with our results. Moreover, in our study, the activities of α-amylase and lipase in the jejunum of pigs fed with glutamine tended to be greater, and, in addition, the activities of lipase in the jejunum of pigs showed significant interactive effects between lactic acid and glutamine. Thus, the increased digestive enzyme activities resulting from dietary supplementation with lactic acid and glutamine may have contributed to the improvement of nutrient digestibility by enhancing pigs’ digestive and absorptive functions.

Another notable discovery in the present study was that the jejunal mucosal *SGLT1*, *GLUT2*, and *PepT1* mRNA abundances were up-regulated following combined supplementation with lactic acid and glutamine. Recent studies have shown that *SGLT1*, *GLUT2*, and *PepT1* are extensively located in the intestinal mucosa, where they serve as the main transporters, respectively, for glucose, glucose, and amino acid [46,47,48]. Therefore, up-regulation of *SGLT1*, *GLUT2*, and *PepT1* mRNA abundances in the jejunal mucosa demonstrated that combined supplementation with lactic acid and glutamine has beneficial effects in enhancing glucose and amino acid absorption by the small intestine after weaning, and, consequently, partially contributes to the rapid growth. Intestinal villus height is directly related to the absorptive surface area of the total luminal villus, and a reduction in the villus could result in inadequate digestive enzyme development [49]. Maintaining or increasing the villus height results in increasing the digestive–absorptive capacity of various nutrients [50]. Conversely, the deepening of crypts means the growth of small intestinal villus epithelial cells is slowed [51]. A lower ratio of villus height to crypt depth is therefore associated with microbial challenges and antigenic components of the feed [52]. In this study, lactic acid exhibited greater villus height and villus height to crypt depth ratio of the jejunum. These results agree with the previous studies that found compound organic acids increased intestinal villus height and villus height to crypt depth [14,45]. Previous studies also confirmed the positive effects of glutamine on intestinal mucosal morphology in weaning piglets, resulting in higher villus height and villus height to crypt depth [33,53,54], which is in line with the present study. Unexpectedly, we found that although there were no significant interactive effect between the combined supplementation of lactate and glutamine in the present article, there was a numerical improvement; we suppose that the positive results observed in the current study may be owing to the following reasons: (1) lactic acid supplementation may promote microecology balance by enhancing gastrointestinal acidity; thus, the acidic environment of the gastrointestine could promote growth and division of intestinal cells and synthetize DNA [44,55]; and (2) glutamine could provide energy and activate the mTOR and ERK 1/2 signal pathways for intestinal epithelial cell proliferation [56,57].

The levels of *IGF-1* and *IGF-1R* in the intestinal epithelium are closely associated with the development of the gastrointestinal tract and mediate intestinal villus enterocytes and the mucosal surface area [58]. *GLP-2* can promote the proliferation of intestinal epithelial cells and inhibit apoptosis [59]. *TGF-β2* plays an important role in the proliferation and differentiation of intestinal epithelial cells [60]. In this study, there were significant interactive effects between lactic acid and glutamine on the expression of *IGF-1*, *IGF-1R*, *TGFβ-2*, and *GLP-2* mRNA levels in pigs, which are consistent with better intestinal morphology in the current study. Moreover, the increased proliferation and maturation of intestinal epithelial cells had beneficial effects on the increase of intestinal digestive and absorptive functions, which is also consistent with the results showing better ATTD of nutrients and digestive enzyme activity in our research.

The intestinal barrier is mainly formed by a layer of epithelial cells associated with tight junctions and is the primary digestive and absorptive site of nutrients. Therefore, the integrity of the intestinal barrier is fundamental to the proper functioning of the epithelial cells and to preventing the entry of pathogenic bacteria that cause inflammation [61,62]. However, stress associated with early weaning in pigs leads to impaired mucosal barrier function [3,63]. Tight junction proteins are the principal determinants of endothelial and epithelial paracellular barrier functions [64]. The tight junction proteins such as *CLDN-2*, *OCLN*, *ZO-1*, and *ZO-2* play a critical role in maintaining intestinal barrier integrity, which efficiently prevents the paracellular diffusion of intestinal bacteria and other antigens across the epithelium [65]. A previous study showed that organic acid supplementation up-regulated *OCLN* and *ZO-1* levels in weaning piglets [66], which is consistent with our results that *OCLN* and *ZO-1* mRNA levels in the jejunum of pigs fed with lactic acid were significantly higher. Furthermore, we noticed that jejunal *OCLN* mRNA abundance was up-regulated by glutamine, which is consistent with the previous study where glutamine increased the jejunal *OCLN* level in weaning piglets [67]. As expected, in our study, expressions of *CLDN-2*, *OCLN*, and *ZO-1* mRNA levels in pigs showed positive interactive effects between lactic acid and glutamine, suggesting that lactic acid and glutamine combined supplementation might have synergistic effects on the functions of tight junction proteins [66,67]. Harmful bacteria (such as pathogenic *Escherichia coli*) have been reported to destabilize and dissociate *ZO-1*, *OCLN*, and *CLDN-1* tight junction complexes, subsequently deteriorating the intestinal barrier function [68], while lactic acid could improve gut microecology balance, and decrease the *Escherichia coli* population [13]. Furthermore, it was reported that lactic acid could exhibit the same intestinal anti-inflammatory effect as certain short-chain fatty acids (butyrate, propionate, acetate, etc.) as a signaling molecule, thus regulating intestinal barrier function [16]. Similarly, anti-inflammatory and antioxidant capacities of glutamine might play an important role in regulating intestinal tight junction levels of weaning piglets [19,32,33]. Collectively, the combined function of lactic acid and glutamine will assist in maintaining better intestinal barrier function in weaning piglets.

A previous study has found that the intestine microbes are associated with nutrient digestion and absorption as well as gut health [69,70]. The balance of beneficial bacteria (such as *Lactobacillus*, *Bifidobacterium*, and *Bacillus*) and harmful bacteria (such as pathogenic *Escherichia coli*) in the gut is related to intestinal morphology and diarrhea [71,72]. Previous studies confirmed the positive effect of organic acid on the intestinal microflora in weaning piglets, resulting in higher *Lactobacillus* and *Bifidobacterium* populations [40,73], which are in line with the effect of lactic acid in the present study. Lactic acid, as a normal acidifier, could decrease enteric pH, which regulates microecology balance [13]. In addition, it is now widely accepted that lactic acid also plays an important role in protective effects against intestinal inflammation, preventing bacterial translocation, as an active signaling metabolite [16]. Unexpectedly, in this study, glutamine supplementation did not exert any influence on the intestinal microflora, which is inconsistent with the previous report that showed that 2% glutamine supplementation could decrease the *Escherichia coli* population in jejunal and colonic digesta as a result of improved intestinal barrier function [74]. This result might be due to differences in weaning age, amount of glutamine, and basal diet [75,76]. Since there were significant interactive effects between lactic acid and glutamine on populations of *Bifidobacterium* in cecal digesta and *Lactobacillus* populations in colonic digesta of the pigs, we suggest that lactic acid and glutamine had synergistic effects on the intestinal microflora of the pigs [13,16,74].

The change in gut microbial composition leads to variation in microbial metabolites. Acetic acid content is negatively correlated with the number of *Escherichia coli* in the intestine, while butyric acid plays a role in maintaining the integrity of the intestinal mucosa and barrier function and inhibiting intestinal inflammation [77]. The VFAs are not only positive in providing energy for intestinal epithelial cells but also promote the formation of cells through stabilizing DNA and repairing damage, thus improving intestinal morphology [55]. In the current study, supplementation of lactic acid significantly increased the content of butyric acid in colonic digesta, which indicated lactic acid promoted the gut microecology balance. Moreover, our present study indicated that there were significant interactive effects between lactic acid and glutamine on acetic acid, butyric acid, and total VFAs in cecal digesta of the pigs. VFAs can inhibit harmful bacteria through increasing intercellular acidity in harmful bacteria, destroying the balance of osmotic pressure in the harmful bacteria, and thus play an important role in regulating the microflora [78]. Therefore, results from the current data indicate that the improved microflora and VFAs induced by the lactic acid and glutamine combination could contribute to a better intestinal environment, thus improving the intestinal health of pigs.

## 5. Conclusions

In summary, the results of the present study indicate that dietary supplementation with lactic acid and glutamine had a positive synergistic effect, and piglets receiving a combination of lactic acid and glutamine had better growth performance, which was associated with improved intestinal function by maintaining intestinal morphology, improving barrier function, and keeping a balance of intestinal microflora. Therefore, our results suggest that lactic acid and glutamine might be a potential feeding additive ensemble to enhance the health and growth of weaning piglets, avoiding weight loss and improving the intestinal health of piglets after weaning in the post-antibiotic era.

## Figures and Tables

**Table 1:** Composition and nutrient content of the experiment basal diets ^1^ (air-dry basis, %).

Item	Days 1–7	Days 8–28
Ingredients, %		
Extruded corn	18.00	25.00
Corn	22.75	36.72
Extruded soybean	4.00	5.00
Dehulled soybean meal	5.00	5.00
Dried whey	15.00	4.00
Soybean protein concentrate	10.00	8.00
Fish meal	5.00	5.00
Spray-dried porcine plasma	6.00	2.00
Sucrose	3.00	3.00
Glucose	5.00	0.00
Coconut oil	4.00	4.00
Calcium carbonate	0.60	0.80
Dicalcium phosphate	0.19	0.21
Choline chloride	0.18	0.18
L-Lysine HCl (98.5%)	0.41	0.36
DL-Methionine	0.20	0.13
L-Threonine	0.13	0.09
L-Tryptophan	0.04	0.01
NaCl	0.20	0.20
Vitamin premix ^2^	0.10	0.10
Mineral premix ^3^	0.20	0.20
Total	100	100
Calculated Nutrients ^4^		
DE (Mcal/kg)	3.65	3.61
CP (%)	21.93	19.22
Ca (%)	0.70	0.68
TP (%)	0.65	0.55
AP (%)	0.47	0.37
STTDP (%)	0.50	0.42
SID-Lysine	1.50	1.35
SID-Met	0.57	0.52
SID-Met + Cys	0.82	0.74
SID-Thr	0.89	0.80
SID-Trp	0.27	0.24

^1^ DE, digestive energy; CP, crude protein; Ca, calcium; TP, total phosphorus; AP, available phosphorus; STTDP, standardized total tract digestibility phosphorus; SID, standardized ileal digestibility. ^2^ Provided per kilogram of diet: vitamin A, 15,000 IU; vitamin D_3_, 1000 IU; vitamin E, 25 IU; vitamin K_3_, 5.0 mg; vitamin B_1_, 2.0 mg; vitamin B_2_, 16 mg; vitamin B_6_, 6.0 mg; vitamin B_12_, 0.03 mg; nicotinic acid, 35 mg; pantothenic acid, 25 mg; folic acid, 2.5 mg; and biotin, 3.3 mg. ^3^ Provided per kilogram of diet: 120 mg Fe (FeSO_4_·7H_2_O); 20 mg Cu (CuSO_4_·5H_2_O); 120 mg Zn (ZnSO_4_·7H_2_O); 15 mg Mn (MnSO_4_·H_2_O); 0.4 mg Se (Na_2_SeO_3_⋅5H_2_O); and 0.3 mg I (KI). ^4^ Values are calculated.

**Table 2:** Sequence of primers used for the real-time quantitative PCR analysis ^1^.

Genes ^1^	Primer Sequences (5′–3′) ^2^	Size (bp)	A_T_ ^3^, °C	Accession Number
*β-actin*	F: TCTGGCACCACACCTTCT	114	57	DQ178122
	R: TGATCTGGGTCATCTTCTCAC			
*SGLT1*	F: AGAAGGGCCCCAAAATGACC	96	58.5	NM_001164021.1
	R: TGTTCACTACTGTCCGCCAC			
*GLUT2*	F: TGGAATCAGCCAACCTGTTT	156	55.8	NM_001097417.1
	R: ACAAGTCCCACCGACATGA			
*PePT1*	F: GCCAAAGTCGTCAAGTGC	100	62.0	NM214347
	R: GGTCAAACAAAGCCCAGA			
*IGF-1*	F: CTGAGGAGGCTGGAGATGTACT	137	58.5	NM_001097417.1
	R: CCTGAACTCCCTCTACTTGTGTTC			
*IGF-1R*	F: GGGATGACGAGAGACATCTATGAG	132	56.8	NM_214172.1
	R: GAAGGACCAGACTCAGACTGC			
*TGF-β2*	F: GAAGCGCATCGAGGCCATTC	162	58.4	NM_214015
	R: GGCTCCGGTTCGACACTTTC			
*CLDN-1*	F: GCCACAGCAAGGTATGGTAAC	140	60.0	NM_001258386.1
	R: AGTAGGGCACCTCCCAGAAG			
*CLDN-2*	F: GCATCATTTCCTCCCTGTT	156	60.0	NM_001161638.1
	R: TCTTGGCTTTGGGTGGTT			
*OCLN*	F: CTACTCGTCCAACGGGAAAG	158	62.0	NM_001163647.2
	R: ACGCCTCCAAGTTACCACTG			
*ZO-1*	F: CAGCCCCCGTACATGGAGA	114	60.0	XM_005659811.1
	R: GCGCAGACGGTGTTCATAGTT			
*ZO-2*	F: ATTCGGACCCATAGCAGACATAG	90	60.0	NM_001206404.1
	R: GCGTCTCTTGGTTCTGTTTTAGC			

^1^ *SGLT1*, sodium–glucose cotransporter 1; *GLUT2*, glucose transporter 2; *PePT1*, peptide transporter 1; *IGF-1*, insulin-like growth factor 1; *IGF-1R*, insulin-like growth factor 1 receptor; *TGF-β2*, transforming growth factor-β2; *CLDN-1*, claudin 1; *CLDN-2*, claudin 2; *OCLN*, occludin; *ZO-1*, zonula occludens 1; *ZO-2*, zonula occludens 2. ^2^ F = forward primer; R = reverse primer. ^3^ A_T_ = annealing temperature.

**Table 3:** Effects of lactic acid, glutamine, and their interactions on growth performance and diarrhea rate in weaning piglets ^1^.

Item ^2^	CON	LS	GS	LGS	SEM ^3^	*p* *	*p* ^+^	*p* ^#^
Initial weight, kg	7.24	7.24	7.24	7.24	0.03	0.99	0.97	0.99
Final weight, kg	14.63	15.26	15.18	15.69	0.18	0.12	0.13	0.15
ADG, g/d	274.02	297.84	296.32	313.23	7.01	0.14	0.12	0.81
ADFI, g/d	484.35 ^b^	528.63 ^a^	517.42 ^a^	546.11 ^a^	11.94	0.04	0.04	0.03
G/F	0.57 ^b^	0.56 ^b^	0.58 ^a^	0.58 ^a^	0.02	0.28	0.01	0.04
Diarrhea rate	0.12	0.06	0.08	0.05	0.01	0.26	0.23	0.11

^a,b^ means different letters in a row differ (*p* < 0.05). ^1^ Means represent *n* = 6. ^2^ CON, the basal diet; LS, supplemented with 2% lactic acid; GS, supplemented with 1% glutamine; LGS, supplemented with 2% lactic acid and 1% glutamine; ADG, average daily gain; ADFI, average daily feed intake; G/F, feed conversion ratio. ^3^ Standard error of the mean. *p* * means lactic acid effect. *p* ^+^ means glutamine effect. *p* ^#^ means lactic acid interactive effect between lactic acid and glutamine.

**Table 4:** Effects of lactic acid, glutamine, and their interactions on apparent total tract digestibility in weaning piglets ^1^.

Item ^2^	CON	LS	GS	LGS	SEM ^3^	*p* *	*p* ^+^	*p* ^#^
Dry matter	85.61	85.62	85.67	85.83	0.04	0.78	0.56	0.97
Crude protein	79.78 ^b^	80.79 ^a^	80.93 ^a^	81.22 ^a^	0.19	0.04	0.03	0.01
Gross energy	85.49	85.58	85.63	85.74	0.05	0.67	0.68	0.88
Crude fat	74.12	75.88	75.69	76.30	0.40	0.42	0.32	0.52
Ash	60.41 ^b^	61.33 ^a^	61.39 ^a^	62.68 ^a^	0.37	0.02	0.03	0.04

^a,b^ means different letters in a row differ (*p* < 0.05). ^1^ Means represent *n* = 6. ^2^ CON, the basal diet; LS, supplemented with 2% lactic acid; GS, supplemented with 1% glutamine; LGS, supplemented with 2% lactic acid and 1% glutamine. ^3^ Standard error of the mean. *p* * means lactic acid effect. *p* ^+^ means glutamine effect. *p* ^#^ means lactic acid interactive effect between lactic acid and glutamine.

**Table 5:** Effects of lactic acid, glutamine, and their interactions on the digestive enzyme activities in the jejunum of weaning piglets ^1^.

Item ^2^	CON	LS	GS	LGS	SEM ^3^	*p* *	*p* ^+^	*p* ^#^
Trypsin, U/mgprot	3336.12	4036.45	3666.65	4012.74	167.12	0.78	0.87	0.78
α-amylase, U/mgprot	785.93 ^b^	1071.12 ^a^	972.62 ^ab^	1064.12 ^a^	48.54	0.01	0.07	0.42
Lipase, U/gprot	361.53 ^b^	526.24 ^a^	484.36 ^ab^	622.75 ^a^	33.67	0.03	0.09	0.04
Sucrase, U/mgprot	83.11	84.12	68.56	68.89	10.02	0.87	0.54	0.67
Maltase, U/gprot	369.19	416.62	378.45	400.02	30.05	0.75	0.67	0.88
Lactase, U/gprot	149.45	167.82	130.72	140.88	18.34	0.32	0.44	0.89

^a,b^ means different letters in a row differ (*p* < 0.05). ^1^ Means represent *n* = 6. ^2^ CON, the basal diet; LS, supplemented with 2% lactic acid; GS, supplemented with 1% glutamine; LGS, supplemented with 2% lactic acid and 1% glutamine. ^3^ Standard error of the mean. *p* * means lactic acid effect. *p* ^+^ means glutamine effect. *p* ^#^ means lactic acid interactive effect between lactic acid and glutamine.

**Table 6:** Effects of lactic acid, glutamine and their interactions on the jejunal morphology of weaning piglets ^1^.

Item ^2^	CON	LS	GS	LGS	SEM ^3^	*p* *	*p* ^+^	*p* ^#^
Villus height, μm	368.73 ^b^	471.09 ^a^	398.67 ^ab^	411.35 ^ab^	15.25	0.04	0.78	0.82
Crypt depth, μm	173.15	172.00	165.53	170.59	4.92	0.70	0.35	0.51
Villus to crypt ratio	2.13 ^b^	2.43 ^a^	2.41 ^a^	2.39 ^ab^	0.10	0.01	0.02	0.52

^a,b^ means different letters in a row differ (*p* < 0.05). ^1^ Means represent *n* = 6. ^2^ CON, the basal diet; LS, supplemented with 2% lactic acid; GS, supplemented with 1% glutamine; LGS, supplemented with 2% lactic acid and 1% glutamine. ^3^ Standard error of the mean. *p* * means lactic acid effect. *p* ^+^ means glutamine effect. *p* ^#^ means lactic acid interactive effect between lactic acid and glutamine.

**Table 7:** Effects of lactic acid, glutamine, and their interactions on the microbial populations (log cfu/g of wet digesta) in cecal and colonic digesta of weaning piglets ^1^.

Item ^2^	CON	LS	GS	LGS	SEM ^3^	*p* *	*p* ^+^	*p* ^#^
Cecal digesta								
*Total bacteria*	10.59	10.76	10.71	10.80	0.03	0.78	0.68	0.85
*Lactobacillus*	6.18 ^b^	6.54 ^ab^	6.55 ^ab^	6.90 ^a^	0.10	0.14	0.15	0.04
*Bifidobacterium*	3.58 ^b^	4.62 ^a^	4.09 ^ab^	4.50 ^ab^	0.13	0.03	0.15	0.56
*Bacillus*	8.01	8.08	8.07	8.20	0.05	0.57	0.34	0.66
*Escherichia coli*	7.32	7.58	7.13	7.25	0.13	0.37	0.39	0.58
Colonic digesta								
*Total bacteria*	11.01	11.13	11.10	11.15	0.02	0.21	0.34	0.43
*Lactobacillus*	7.18 ^b^	7.61 ^a^	7.23 ^ab^	7.63 ^ab^	0.08	0.01	0.56	0.23
*Bifidobacterium*	4.80 ^b^	5.23 ^ab^	5.05 ^ab^	5.68 ^a^	0.13	0.23	0.35	0.03
*Bacillus*	7.61	7.87	7.79	7.55	0.18	0.50	0.48	0.89
*Escherichia coli*	8.41	8.50	8.44	8.51	0.04	0.23	0.40	0.33

^a,b^ means different letters in a row differ (*p* < 0.05). ^1^ Means represent *n* = 6. ^2^ CON, the basal diet; LS, supplemented with 2% lactic acid; GS, supplemented with 1% glutamine; LGS, supplemented with 2% lactic acid and 1% glutamine. ^3^ Standard error of the mean. *p* * means lactic acid effect. *p* ^+^ means glutamine effect. *p* ^#^ means lactic acid interactive effect between lactic acid and glutamine.

**Table 8:** Effects of lactic acid, glutamine, and their interactions on the intestinal microbial metabolites (μmol/g of wet digesta) in the cecal and colonic digesta of weaning piglets ^1^.

Item ^2^	CON	LS	GS	LGS	SEM ^3^	*p* *	*p* ^+^	*p* ^#^
Cecal digesta								
Acetic acid	25.16 ^b^	26.49 ^ab^	27.09 ^ab^	31.49 ^a^	1.04	0.72	0.56	0.02
Propanoic acid	9.93	11.19	11.46	14.34	0.80	0.37	0.72	0.20
Butyric acid	4.67 ^b^	5.78 ^ab^	7.06 ^ab^	8.83 ^a^	0.59	0.21	0.32	0.03
TVFAs	39.76 ^b^	43.46 ^ab^	45.61 ^ab^	54.66 ^a^	2.17	0.12	0.21	0.01
Colonic digesta								
Acetic acid	18.12	19.30	21.85	17.18	1.04	0.81	0.50	0.58
Propanoic acid	7.25	8.16	9.04	8.16	0.40	0.32	0.31	0.82
Butyric acid	4.65 ^b^	6.61 ^a^	5.89 ^ab^	5.78 ^ab^	0.34	0.02	0.72	0.90
TVFAs	30.03	34.08	36.78	32.59	1.42	0.68	0.47	0.90

^a,b^ means different letters in a row differ (*p* < 0.05). ^1^ Means represent *n* = 6. ^2^ CON, the basal diet; LS, supplemented with 2% lactic acid; GS, supplemented with 1% glutamine; LGS, supplemented with 2% lactic acid and 1% glutamine; TVFAs, total volatile fatty acids. ^3^ Standard error of the mean. *p* * means lactic acid effect. *p* ^+^ means glutamine effect. *p* ^#^ means lactic acid interactive effect between lactic acid and glutamine.

## Data Availability

The datasets used to support the findings of this study are available from the corresponding author upon request.
